# Supplementary material for: Evaluation of Vaccine Strategies among Healthcare Workers during COVID-19 Omicron Outbreak in Taiwan
Source: Vaccines (Basel). 2024 Sep 17;12(9):1057. doi: 10.3390/vaccines12091057 (PMC11435596; doi:10.3390/vaccines12091057)
Supplement: Supplementary file 1 [file vaccines-12-01057-s001.zip › vaccines-3189747-supplementary.pdf]

**Supplementary Materials**

**Table S1**. Rates (%) of solicited local and systemic adverse events occurred within seven days of immunization with Covid-19 vaccines of different platforms.

| | Vaccine | Severity grading/Rates % | | | |
|---|---|---|---|---|---|
| | | Grade 1 | Grade 2 | Grade 3 | No event |
| First dose | | | | | |
| Pain | AstraZeneca | 60.8 | 19.7 | 1.01 | 18.5 |
| | Moderna | 73.9 | 13.9 | 1.74 | 10.4 |
| Erythema | AstraZeneca | 11.6 | 5.22 | 0.34 | 82.8 |
| | Moderna | 23.5 | 1.74 | 0.87 | 73.9 |
| Swelling | AstraZeneca | 23.6 | 5.56 | 0.17 | 70.7 |
| | Moderna | 37.4 | 9.57 | 0 | 53 |
| Fever | AstraZeneca | 35.5 | 10.8 | 2.02 | 51.7 |
| | Moderna | 3.48 | 0.87 | 0 | 95.7 |
| Headache | AstraZeneca | 28.5 | 22.2 | 3.03 | 46.3 |
| | Moderna | 12.2 | 7.83 | 0 | 80 |
| Malaise | AstraZeneca | 34.3 | 33.5 | 6.57 | 25.6 |
| | Moderna | 32.2 | 6.96 | 1.74 | 59.1 |
| Myalgia | AstraZeneca | 32.3 | 23.7 | 4.04 | 39.9 |
| | Moderna | 15.7 | 1.74 | 0.87 | 81.7 |
| Anorexia | AstraZeneca | 11.1 | 4.04 | 0.34 | 84.5 |
| | Moderna | 6.09 | 0.87 | 0 | 93 |
| Diarrhea | AstraZeneca | 9.6 | 4.21 | 0.51 | 85.7 |
| | Moderna | 6.96 | 2.61 | 0 | 90.4 |
| Second dose | | | | | |
| Pain | AstraZeneca | 47.3 | 2.66 | 0 | 50 |
| | Moderna | 45 | 38.3 | 8.81 | 7.9 |
| Erythema | AstraZeneca | 5.85 | 0.27 | 0 | 93.9 |
| | Moderna | 19.5 | 7.9 | 1.82 | 70.8 |
| Swelling | AstraZeneca | 12.5 | 0.53 | 0 | 87 |
| | Moderna | 35.6 | 11.6 | 1.52 | 51.4 |
| Fever | AstraZeneca | 4.26 | 0.53 | 0 | 95.2 |
| | Moderna | 41.6 | 12.8 | 1.52 | 44.1 |
| Headache | AstraZeneca | 11.7 | 2.93 | 1.33 | 84 |
| | Moderna | 31 | 22.49 | 3.95 | 42.55 |
| Malaise | AstraZeneca | 26.33 | 4.79 | 1.06 | 67.82 |
| | Moderna | 38.3 | 36.17 | 6.99 | 18.54 |
| Myalgia | AstraZeneca | 14.6 | 2.13 | 0.27 | 83 |

| | | | | | |
|---|---|---|---|---|---|
| | Moderna | 30.09 | 26.14 | 6.69 | 37.08 |
| Anorexia | AstraZeneca | 3.99 | 0 | 0 | 96 |
| | Moderna | 12.2 | 2.13 | 0 | 85.7 |
| Diarrhea | AstraZeneca | 3.72 | 0.27 | 0 | 96 |
| | Moderna | 11.3 | 3.34 | 0.91 | 84.5 |
| Third dose | | | | | |
| Pain | Medigen | 60.4 | 5.66 | 0 | 34.0 |
| | Moderna | 62.8 | 21.3 | 1.65 | 14.3 |
| | Pfizer-BioNTech | 63.8 | 18.8 | 0 | 17.4 |
| Erythema | Medigen | 7.55 | 0 | 0 | 92.5 |
| | Moderna | 11.0 | 3.85 | 0.37 | 84.8 |
| | Pfizer-BioNTech | 10.1 | 2.90 | 0 | 87.0 |
| Swelling | Medigen | 18.9 | 0 | 0 | 81.1 |
| | Moderna | 27.3 | 6.78 | 0.55 | 65.4 |
| | Pfizer-BioNTech | 8.62 | 9.76 | 0 | 11.1 |
| Fever | Medigen | 3.77 | 0 | 0 | 96.2 |
| | Moderna | 15.4 | 2.56 | 0.37 | 81.7 |
| | Pfizer-BioNTech | 14.5 | 1.45 | 0 | 84.1 |
| Headache | Medigen | 5.66 | 0 | 0 | 94.3 |
| | Moderna | 21.1 | 8.61 | 1.47 | 68.9 |
| | Pfizer-BioNTech | 26.1 | 5.80 | 0 | 68.1 |
| Malaise | Medigen | 18.9 | 0 | 0 | 81.1 |
| | Moderna | 36.5 | 12.8 | 2.38 | 48.4 |
| | Pfizer-BioNTech | 36.2 | 15.9 | 0 | 47.8 |
| Myalgia | Medigen | 7.55 | 0 | 0 | 92.5 |
| | Moderna | 24.0 | 11.9 | 1.65 | 62.5 |
| | Pfizer-BioNTech | 29.0 | 10.1 | 0 | 60.9 |
| Anorexia | Medigen | 0 | 0 | 0 | 100.0 |
| | Moderna | 6.41 | 1.65 | 0.18 | 91.8 |
| | Pfizer-BioNTech | 8.70 | 1.45 | 0 | 89.9 |
| Diarrhea | Medigen | 3.77 | 0 | 0 | 96.2 |
| | Moderna | 5.13 | 1.47 | 0.18 | 93.2 |
| | Pfizer-BioNTech | 5.80 | 1.45 | 0 | 92.8 |
| Fourth dose | | | | | |
| Pain | Medigen | 44.83 | 3.45 | 0 | 51.72 |
| | Novavax | 64.71 | 0 | 0 | 35.29 |
| | Moderna | 64.85 | 18.79 | 0.61 | 15.76 |
| | Moderna_BA1 | 66.67 | 21.21 | 0 | 12.12 |

| | | | | | |
|---|---|---|---|---|---|
| | Pfizer-BioNTech | 58.7 | 15.22 | 2.17 | 23.91 |
| Erythema | Medigen | 0 | 0 | 0 | 100 |
| | Novavax | 0 | 0 | 5.88 | 94.12 |
| | Moderna | 3.64 | 5.45 | 1.21 | 89.7 |
| | Moderna_BA1 | 3.03 | 0 | 6.06 | 90.91 |
| | Pfizer-BioNTech | 6.52 | 2.17 | 6.52 | 84.78 |
| Swelling | Medigen | 3.45 | 0 | 3.45 | 93.1 |
| | Novavax | 5.88 | 0 | 0 | 94.12 |
| | Moderna | 11.52 | 8.48 | 9.09 | 70.91 |
| | Moderna_BA1 | 6.06 | 0 | 3.03 | 90.91 |
| | Pfizer-BioNTech | 8.7 | 13.04 | 8.7 | 69.57 |
| Fever | Medigen | 0 | 0 | 0 | 100 |
| | Novavax | 5.88 | 0 | 0 | 94.12 |
| | Moderna | 8.48 | 1.21 | 0 | 90.3 |
| | Moderna_BA1 | 6.06 | 0 | 0 | 93.94 |
| | Pfizer-BioNTech | 10.87 | 2.17 | 0 | 86.96 |
| Headache | Medigen | 6.9 | 3.45 | 0 | 89.66 |
| | Novavax | 11.76 | 5.88 | 5.88 | 76.47 |
| | Moderna | 11.52 | 6.06 | 0.61 | 81.82 |
| | Moderna_BA1 | 18.18 | 12.12 | 0 | 69.7 |
| | Pfizer-BioNTech | 13.04 | 6.52 | 0 | 80.43 |
| Malaise | Medigen | 6.9 | 0 | 0 | 93.1 |
| | Novavax | 23.53 | 5.88 | 5.88 | 64.71 |
| | Moderna | 23.03 | 10.91 | 1.21 | 64.85 |
| | Moderna_BA1 | 27.27 | 21.21 | 0 | 51.52 |
| | Pfizer-BioNTech | 28.26 | 13.04 | 0 | 58.7 |
| Myalgia | Medigen | 6.9 | 3.45 | 0 | 89.66 |
| | Novavax | 11.76 | 5.88 | 0 | 82.35 |
| | Moderna | 12.12 | 8.48 | 1.82 | 77.58 |
| | Moderna_BA1 | 33.33 | 6.06 | 0 | 60.61 |
| | Pfizer-BioNTech | 26.09 | 8.7 | 0 | 65.22 |
| Anorexia | Medigen | 3.45 | 0 | 0 | 96.55 |
| | Novavax | 0 | 0 | 0 | 100 |
| | Moderna | 1.82 | 2.41 | 0 | 93.94 |
| | Moderna_BA1 | 6.06 | 3.03 | 0 | 90.91 |
| | Pfizer-BioNTech | 4.35 | 0 | 0 | 95.65 |
| Diarrhea | Medigen | 0 | 0 | 0 | 100 |
| | Novavax | 0 | 0 | 0 | 100 |

| | | | | |
|---|---|---|---|---|
| Moderna | 2.42 | 1.82 | 0 | 95.76 |
| Moderna_BA1 | 6.06 | 0 | 0 | 93.64 |
| Pfizer-BioNTech | 10.87 | 0 | 0 | 89.13 |

**Table S2.** Grading of severity in subjects with adverse events post immunization.

| Adverse events | Descriptions |
|---|---|
| General severity | |
| 0 | Normal |
| 1 | Mild, no interference with daily activity |
| 2 | Moderate, mild to moderate interference of daily activity, minimal medical intervention/therapy required (i.e. use of non-opioid treatment, vomitus no more than 2 times per day, diarrhea episodes no more than 3 times per day) |
| 3 | Severe, considerable interference with the daily activity; medical intervention/therapy required (i.e. use of opioid treatment, required anti-emetic agents for vomiting, diarrhea episodes more than five times per day) |
| 4 | Life-threatening; requiring hospitalization |
| Fever severity | |
| 0 | < 38˚C |
| 1 | 38 – < 39˚C |
| 2 | 39 – < 40˚C |
| 3 | ≥ 40˚C |
| Erythema severity | |
| 0 | 0 cm |
| 1 | < 5 cm |
| 2 | 5 – < 10 cm |
| 3 | > 10 cm |
| Swelling severity | |
| 0 | 0 cm |
| 1 | < 5 cm |
| 2 | 5 – < 10 cm |
| 3 | > 10 cm |

**Table S3.** List of the non-solicited adverse events (AEs) that occurred within 7 days of immunization against COVID-19. The number in the parenthesis following the AE indicates the number of subjects reporting the AE.

| | |
|---|---|
| AstraZeneca Dose 1 (N = 594) | **dizziness (16, 2.69%), chillness (13, 2.19%), rash (11, 1.85%), abdominal pain (8, 1.35%), palpitation (6, 1.01%), joint pain (6, 1.01%), itching of injection site (6, 1.01%),** dyspnea (5), sore throat (5), sleepy (4), poor appetite (4), Lymphadenitis (4), sweating (4), menstrual cycle change (3), chest tightness/pain (3), ecchymosis (3), itching of trunk (3), dysgeusia (3), running nose (3), periorbital swelling (3), tinnitus (2), thirsty (1), feel pain of internal organs (1), gum swelling (1), tension at head (1), toothache (1), weakness (1), buttock pain (1), interchangeable warm and cold sensation over limbs (1), generalized sting pain (1), parotiditis (1), hypoglycemia (1), convulsion (1), hypertension (1), scar formation of injection site (1), soreness of the injected arm (1), frozen shoulder (1), shoulder pain (1), increased appetite (1), mania (1), eye pain (1), conjunctivitis (1), oral ulcer (1), hand numbness (1), face numbness (1), dysesthesia (1), dry eyes (1), epistaxis (1) |
| AstraZeneca Dose 2 (N = 376) | **Dizziness (6, 1.60%), abdominal pain (5, 1.33%),** rash (3), hypertension (3), palpitation (2), limb weakness (2), itching of injection site (2), increased appetite (2), generalized itching (2), limb soreness (1), soreness of injection site (1), frozen shoulder (1), periorbital swelling (2), dry eyes (1), sore throat (2), menstrual cycle change (1), flank pain (1), joint pain (1), migraine (1), periorbital pain (1) |
| Moderna Dose 1 (N = 115) | **itching of injection site (23, 20.0%), lymphadenitis (9, 7.83%), dizziness (4, 3.48%), joint pain (3, 2.60%), palpitation (2, 1.74%), sore throat (2, 1.74%), abdominal pain (2, 1.74%), generalized rash (2, 1.74%), rash of injection site (2, 1.74%),** dysesthesia (1), generalized itching (1), thirsty (1), toothache (1), numbness of injection site (1), running nose (1), chest tightness (1), red eyes (1), dry eyes (1), decreased visual acuity (1), menstrual cycle change (1), flank pain (1), generalized heat sensation (1), dyspnea (1), frozen shoulder (1), sneezing (1). |

| | |
|---|---|
| Moderna Dose 2 (N = 329) | **Itching of injection site (17, 5.17%), dizziness (15, 4.56%), chills (11, 3.34%), palpitation (10, 3.04%), lymphadenitis (7, 2.13%), rash (7, 2.13%), joint pain (7, 2.13%), chest pain/tightness (7, 2.13%),** abdominal pain/distension (3), stuffy nose (3), sore throat (3), general soreness (3), limb pain (3), generalized itching (2), dry eyes (2), sting pain (2), tinnitus (2), poor appetite (2), dyspnea (2), menstrual cycle change (2), flank pain (2), oral ulcer (1), shoulder pain (2), sweating (2), insomnia (1), ecchymosis (1), tongue numbness (1), finger numbness (1), limb numbness (1), running nose (1), urine urgency (1), ear pain (1), hypotension (1), interchangeable warm and cold sensation (1), limb weakness (1), increased appetite (1), eye pain (1) |
| Moderna Dose 3 (N = 546) | **Lymphadenitis (20, 3.66%), chills (19, 3.48%), dizziness (10, 1.83%), palpitation (6, 1.10%),** sleepy (4), rash (4), chest pain (3), limb pain (3), generalized itching (3), bone pain (2), itching of injection site (2), dry eyes (2), joint pain (2), sweating (2), ecchymosis (2), generalized soreness (1), tinnitus (1), dyspnea (1), hoarseness (1), dry mouth (1), toothache (1), weakness (1), cold sensation over limbs (1), menstrual cycle change (1), interchangeable heat and cold sensation, zoster (1), periorbital swelling (1), flank pain (1), sore throat (1), limb numbness (1), stuffy nose (1) |
| Moderna Dose 4 (N = 165) | **Lymphadenitis (3),** limb pain (1), general itching (1), bone soreness (1), urticaria (1), flank pain (1), injection site itching (1), sleepy (1), abdominal pain (1) |
| Pfizer-BioNTech Dose 3 (N = 69) | **Lymphadenitis (5, 7.25%), itching sensation of injection site (2, 2.90%), chills (2, 2.90%),** dizziness (1), palpitation (1), bone pain (1), joint pain (1), chest pain (1) |
| Pfizer/BioNTech Dose 4 (N = 46) | Lymphadenitis (1), limb pain (1), general itching (1), dyspnea (1), palpitation (1) |
| Medigen Dose 3 (N = 53) | Dizziness (1), limb pain (1), increased appetite (1) |

| | |
|---|---|
| Medigen Dose 4 (N = 29) | Bone soreness (1) |
| Moderna BA1 Dose 4 (N = 33) | Lymphadenitis (1), abdominal pain (1), eye pain (1) |
| Novavax Dose 4 (N = 17) | Toothache (1), ecchymosis (1), stuffy nose (1), acnes (1), cough (1) |

**Table S4.** Geometric mean titers and 95% confidence limits of neutralizing antibody against SARS-CoV-2 in different occasions in individuals on different immunization schedules.

| | GMT | 95% CL-Lower | 95% CL-Upper |
|---|---|---|---|
| Pre first vaccine dose | | | |
| AAG (n=28) | 6.15 | 6.15 | 6.15 |
| AAM(n=267) | 6.15 | 6.15 | 6.15 |
| AAB(n=53) | 6.24 | 6.15 | 6.34 |
| AMG(n=16) | 6.15 | 6.15 | 6.15 |
| AMM(n=178) | 6.15 | 6.15 | 6.15 |
| AMB(n=13) | 6.38 | 6.03 | 6.76 |
| MMG(n=9) | 6.15 | 6.15 | 6.15 |
| MMM(n=101) | 6.15 | 6.15 | 6.15 |
| Pre second vaccine dose | | | |
| AAG (n=28) | 19.3 | 11.6 | 32.1 |
| AAM(n=267) | 20 | 16.9 | 23.7 |
| AAB(n=53) | 23.1 | 15.4 | 34.8 |
| AMG(n=16) | 27.5 | 11.7 | 64.5 |
| AMM(n=178) | 16.9 | 14 | 20.4 |
| AMB(n=13) | 19.3 | 7.92 | 46.9 |
| MMG(n=9) | 327.3 | 225.9 | 474.4 |
| MMM(n=101) | 231.8 | 190.2 | 282.4 |
| 30 days post second vaccine dose ( | | | |
| AAG (n=28) | 63.1 | 33.8 | 117.8 |
| AAM(n=267) | 55.4 | 46.7 | 65.6 |
| AAB(n=53) | 61.5 | 42.5 | 88.8 |
| AMG(n=16) | 663.6 | 454 | 969.7 |
| AMM(n=178) | 648.2 | 595.7 | 705.3 |
| AMB(n=13) | 650.4 | 541.9 | 780.8 |
| MMG(n=9) | 1379.9 | 676.2 | 2815.9 |
| MMM(n=101) | 1113.6 | 972.5 | 1275.2 |

| | | | |
|---|---|---|---|
| Pre third vaccine dose | | | |
| AAG (n=28) | 11.6 | 7.76 | 17.2 |
| AAM(n=267) | 10 | 9.07 | 11.1 |
| AAB(n=53) | 8.05 | 6.94 | 9.34 |
| AMG(n=16) | 139 | 93.6 | 206.5 |
| AMM(n=178) | 122.6 | 107 | 140.5 |
| AMB(n=13) | 169.8 | 131.8 | 218.8 |
| MMG(n=9) | 210.6 | 165.9 | 267.3 |
| MMM(n=101) | 243.4 | 212.5 | 278.8 |
| 30 days post third vaccine dose | | | |
| AAG (n=28) | 302.2 | 239.5 | 381.3 |
| AAM(n=267) | 698.5 | 665.5 | 733.1 |
| AAB(n=53) | 618.9 | 549.6 | 696.9 |
| AMG(n=16) | 269 | 221.1 | 327.3 |
| AMM(n=178) | 740.4 | 699 | 784.3 |
| AMB(n=13) | 718.8 | 577.6 | 894.6 |
| MMG(n=9) | 384.8 | 314.3 | 471 |
| MMM(n=101) | 915.3 | 879.9 | 952.1 |
| Pre fourth vaccine dose | | | |
| Medigen (n=29) | 192.1 | 140.4 | 263 |
| Novavax (n=17) | 251.4 | 159.9 | 395.2 |
| Moderna (n=165) | 200.7 | 165.8 | 243 |
| Moderna BA1 (n=33) | 265.2 | 133.1 | 528.5 |
| BioNTech (n=46) | 213.9 | 169.4 | 270.1 |
| 30 days post fourth vaccine dose | | | |
| Medigen (n=29) | 333.3 | 261.4 | 425 |
| Novavax (n=17) | 666.7 | 437.6 | 1015.8 |
| Moderna (n=165) | 971.8 | 913.5 | 1033.7 |
| Moderna BA1 (n=33) | 1757.9 | 1378.8 | 2241.2 |
| BioNTech (n=46) | 872.8 | 745.3 | 1022 |

**Table S5.** COVID-19 diagnosis by the review of medical records, questionnaires, and the anti-N antibody measurement.

| | | Medical records and questionnaire | | | |
|---|---|---|---|---|---|
| | | Negative | Positive | Not available | Total |
| Anti-N antibody | Negative | 198 (A) | 179 (B) [#] | 123 (C) | 500 |
| | Positive | 21 (D) | 125 (E) | 6 (F) | 152 |
| | Not available | 1 (G) | 4 (H) | 53 (I) | 58 |
| | Total | 220 | 308 | 182 | 710 |

COVID-19 diagnosis = B+D+E+F+H = 335

Absence of COVID-19 = A+C+G = 322

Not available (missing data) = I = 53

[#] The negative results of anti-N in subjects with COVID-19 were due to the fact that the blood samples were collected only before and 30 days after vaccination, but not at the time of diagnosis.

**Table S6.** Cox regression analysis of factors associated with absence of COVID-19 in 255 healthcare workers having the fourth dose of vaccine against SARS-CoV-2.

| Parameter | Parameter estimate | Standard error | Chi-square | Pr > ChiSq | Hazard ratio | 95% HR confidence limits | |
|---|---|---|---|---|---|---|---|
| Dose 4 (vs. Pfizer-BioNTech) | | | | | | | |
| Medigen | -0.40093 | 0.49158 | 0.6652 | 0.4147 | 0.670 | 0.256 | 1.755 |
| Moderna | -0.29615 | 0.30967 | 0.9146 | 0.3389 | 0.744 | 0.405 | 1.365 |
| Moderna_BA1 | -1.04188 | 0.52452 | 3.9456 | 0.0470 | 0.353 | 0.126 | 0.986 |
| Novavax | 0.18593 | 0.53565 | 0.1205 | 0.7285 | 1.204 | 0.421 | 3.441 |
| Dose 1-3 (vs. MMM) | | | | | | | |
| AAB | -0.20979 | 0.48394 | 0.1879 | 0.6646 | 0.811 | 0.314 | 2.093 |
| AAG | -0.66248 | 0.80901 | 0.6706 | 0.4129 | 0.516 | 0.106 | 2.517 |
| AAM | 0.01542 | 0.34767 | 0.0020 | 0.9646 | 1.016 | 0.514 | 2.007 |
| AMB | -0.40108 | 0.80129 | 0.2505 | 0.6167 | 0.670 | 0.139 | 3.220 |

| | | | | | | | |
|---|---|---|---|---|---|---|---|
| AMG | -0.23421 | 0.84366 | 0.0771 | 0.7813 | 0.791 | 0.151 | 4.134 |
| AMM | -0.27814 | 0.37671 | 0.5452 | 0.4603 | 0.757 | 0.362 | 1.584 |
| MMB | 1.02329 | 1.13355 | 0.8149 | 0.3667 | 2.782 | 0.302 | 25.662 |
| MMG | 0.46577 | 1.13636 | 0.1680 | 0.6819 | 1.593 | 0.172 | 14.776 |
| Female gender | 0.14587 | 0.24646 | 0.3503 | 0.5540 | 1.157 | 0.714 | 1.876 |
| Age, in years | 0.01051 | 0.00986 | 1.1344 | 0.2868 | 1.011 | 0.991 | 1.030 |
